# Premature mortality due to noncommunicable diseases in Brazilian capitals: redistribution of garbage causes and evolution by social deprivation strata

**DOI:** 10.1590/1980-549720230002.supl.1

**Published:** 2023-04-21

**Authors:** Deborah Carvalho Malta, Renato Azeredo Teixeira, Laís Santos de Magalhães Cardoso, Juliana Bottoni de Souza, Regina Tomie Ivata Bernal, Pedro Cisalpino Pinheiro, Crizian Saar Gomes, Alastair Leyland, Ruth Dundas, Maurício Lima Barreto

**Affiliations:** I Universidade Federal de Minas Gerais, School of Nursing, Department of Maternal and Child Nursing and Public Health – Belo Horizonte (MG), Brazil.; II Universidade Federal de Minas Gerais, School of Medicine, Graduate Program in Public Health – Belo Horizonte (MG), Brazil.; III Universidade Federal de Minas Gerais, School of Nursing, Graduate Program in Nursing – Belo Horizonte (MG), Brazil.; IV Universidade Federal de Minas Gerais, School of Nursing, Observatório de Doenças e Agravos Não Transmissíveis – Belo Horizonte (MG), Brazil.; V University of Glasgow, MRC/CSO Social & Public Health Sciences Unit – Glasgow, United Kingdom.; VI Fundação Oswaldo Cruz, Centro de Integração de Dados e Conhecimentos para Saúde – Salvador (BA), Brazil.; VII Universidade Federal da Bahia, Instituto de Saúde Coletiva – Salvador (BA), Brazil.

**Keywords:** Noncommunicable diseases, Mortality, premature, Mortality registries, Health status disparities, Small-area analysis

## Abstract

**Objective:**

To analyze premature mortality due to noncommunicable chronic diseases (NCDs) in Brazilian capitals and the Federal District (DF) after redistribution of garbage causes and the temporal evolution according to social deprivation strata in the 2010 to 2012 and 2017 to 2019 triennia.

**Methods:**

Corrections were applied to the Mortality Information System (*Sistema de Informação sobre Mortalidade* – SIM) data such as the redistribution of garbage codes (GC). Premature mortality rates due to NCDs were calculated and standardized by age. The differences among NCDs mortality rates were analyzed according to the Brazilian Deprivation Index (*Índice Brasileiro de Privação* – IBP) categories and between the three-year periods.

**Results:**

In the capitals as a whole, rates increased between 8 and 12% after GC redistribution and the greatest increases occurred in areas of high deprivation: 11.9 and 11.4%, triennia 1 and 2, respectively. There was variability between the capitals. There was a reduction in rates in all strata of deprivation between the three-year periods, with the greatest decrease in the stratum of low deprivation (-18.2%) and the lowest in the stratum of high deprivation (-7.5%).

**Conclusion:**

The redistribution of GC represented an increase in mortality rates, being higher in the strata of greater social deprivation. As a rule, a positive gradient of mortality was observed with increasing social deprivation. The analysis of the temporal evolution showed a decrease in mortality from NCDs between the triennia, especially in areas of lower social deprivation.

## INTRODUCTION

Noncommunicable Chronic Diseases (NCDs) represent the major cause of morbidity and mortality in Brazil and worldwide^1,2^. It is estimated that they are responsible for 41 million deaths worldwide per year and, of which, 15 million are considered premature because they affect individuals aged between 30 and 69 years old^3,4^. In Brazil, in 2019, general mortality from NCDs corresponded to about 76% of all deaths, with 66.1% being premature deaths^5^.

NCDs impose a significant and growing burden on health systems and the general economy of countries^6^. It is estimated that economic growth is reduced by up to 0.5% for each 10% increase in NCD mortality. Thus, NCDs stand out among the main global threats to economic development, as well as to the health and well-being of populations, thus, monitoring them is paramount^7^.

Social determinants strongly contribute to the increase and severity of NCDs^8^. These diseases and their risk factors have a more negative impact on socioeconomically vulnerable populations, since they have less access to health services and environments considered healthy^9^. This deficit, in turn, reduces opportunities for prevention and health promotion among these populations^10–12^.

Several national and global commitments were established with the objective to reduce NCDs, including the Strategic Action Plan to Tackle NCDs in Brazil 2011–2022^13^, the World Health Organization (WHO) Global Action Plan for the Prevention and Control of NCDs^14^, and the United Nation's 2030 Agenda for the Sustainable Development. The UN included the goal of reducing NCDs by one third by 2030^15^. Monitoring inequalities in the distribution of mortality due to NCDs becomes important in the context of the Sustainable Development Goals (SDG)^15^.

In Brazil, the Mortality Information System (*Sistema de Informação sobre Mortalidade* – SIM) makes it possible to monitor mortality patterns from these causes and their evolution over time^16^, constituting an essential source of national data. However, problems related to the completion of death certificates (DC) — instruments that feed the SIM — result in compromised quality of mortality information. Although there has been an improvement in SIM coverage and DC completion in recent decades, it is still necessary to adopt methods to correct underreporting of deaths, missing data, and excessive recording of garbage causes (GC) — which cannot be considered basic causes of death — in order to minimize biases in mortality estimates^2^.

In addition to monitoring premature mortality from NCDs on a national scale, it is necessary to monitor the evolution of this event in small areas — geographic or relative to population subgroups. Reliable estimates for small areas enable a more detailed situational diagnosis and contribute to the identification of health inequities, in addition to supporting the planning and prioritization of public health actions^17^.

This is the first study that applies the redistribution method of GC to generate mortality estimates in Brazilian capitals and in different strata of deprivation, seeking to qualify the SIM database.

In view of the above, this study aimed to analyze premature mortality due to NCDs in Brazilian capitals and the Federal District (*Distrito Federal* – DF) after redistribution of GC, and the temporal evolution according to social deprivation strata in the three-year periods 2010 to 2012 and 2017 to 2019.

## METHODS

### Study design, population and period

This is a descriptive epidemiological study. Estimates of premature mortality from NCDs in Brazilian capitals and in the Federal District for the three-year periods 2010 to 2012 and 2017 to 2019 were analyzed.

### Indicators and data source

#### Premature mortality rate from NCD

For the calculation of mortality rates, data on deaths from the Mortality Information System were used. The database was provided by the Ministry of Health at the request of the researchers and contains information on the census tracts of the deceased's homes. Population estimates, available on the Datasus^18^ website, were used as a denominator in the calculation of mortality rates.

According to the WHO definition, premature deaths from NCDs were considered, which include deaths due to cardiovascular diseases, chronic respiratory diseases, neoplasms, and diabetes mellitus in the population aged between 30 and 69 years old^4,14^.

The mortality rates calculated for each capital and year were standardized by age, using the direct method^18^. In order to eliminate the effects of differences between age structures, the Brazilian population from the 2010 demographic census was adopted as the standard population^18,19^.

In calculating the rates, the numerator comprised the number of deaths estimated by age-standardized rates, and the denominator included the total population of the triennia for each capital.

#### Social deprivation index

The Brazilian Deprivation Index (*Índice Brasileiro de Privação* – IBP), launched in 2020 and developed by researchers from the Center for the Integration of Data and Knowledge for Health (*Centro de Integração de Dados e Conhecimentos para Saúde* – CIDACS) and the University of Glasgow, in Scotland, is a proposal to measure social inequalities in Brazil on the scale of census tracts. IBP calculation considered the combination of z-scores of three deprivation indicators on the census tracts scale: percentage of households with income lower than half a minimum wage; percentage of illiterate individuals aged seven years old or older; and percentage of individuals with inadequate access to water, sanitation, waste collection, and no toilet^20^.

IBP generates a final score in which the lowest value represents the area with the least deprivation, while the highest value represents the area with the greatest deprivation^20,21^. It should be noted that the 65,687 census tracts of the 26 capitals and the DF are home to 45,980,851 people, corresponding to 22% of all census tracts and 24% of the Brazilian population. There was a greater concentration of deprivation/poverty in capitals in the North and Northeast, and absence of census tracts with high deprivation in the South, Southeast, and Central-West regions. Due to this unequal distribution of quintiles of census tracts with high deprivation in the capitals, only three deprivation strata were used in the current study: The “low deprivation” stratum, which corresponds to quintile 1; “medium deprivation”, which corresponds to quintile 2; and “high deprivation”, corresponding to quintiles 3, 4, and 5.

### Treatment of death data prior to the calculation of mortality rates

Considering the heterogeneity of the SIM quality in the country and over time, methods were applied to standardize the quality of the capitals death databases, which addressed the missing data and the quality of the definition of the underlying causes of death.

The first stage consisted of treating the missing data, through the proportional redistribution of ignored and blank data, according to year, age, gender, and place of residence^22^. Subsequently, the redistribution of GC was carried out. This term defines a group of causes that cannot be considered basic causes of death, that is, it refers to ill-defined or unspecific causes that are, therefore, of little use for public health, since they make it difficult to identify the real diseases and injuries that caused death^22^. Thus, GC should be detected and redistributed among cause-specific deaths, in order to improve the validity of mortality analyses^22,23^.

In the GC redistribution process, firstly, the definition of the groups of causes was considered according to the list of the Global Burden of Disease (GBD) 2017^24^, which includes other causes in addition to the chapter of ill-defined causes of the International Classification of Diseases and Related Health Problems (ICD-10)^25^. An analysis of the codes on this list was performed to identify which GC are specifically related to NCDs^22^. Deaths whose underlying causes, relative to the groups considered in this study, correspond to codes specified in the ICD-10 and defined by the GBD 2017 study were classified as target causes, or causes to which the GC will be redistributed. Subsequently, redistributions were made according to GC levels and their respective targets^22^. In addition to the proportional redistribution process, the study considered the results of GC investigations initiated in 2016^26^.

### Analytical procedures

The 95% confidence intervals of premature mortality rates due to NCDs were calculated. Comparisons using relative percentage variation (RPV) were made between the two triennia (2010 to 2012 and 2017 to 2019) and between rates with data before and after GC redistribution, which aims to show the impact of the method on the risk of death estimated from SIM data. All analyses were performed according to capitals and respective IBP categories.

The census tracts, referring to the places of residence of the deceased individuals, were used as a key variable to assign the deprivation classification of these individuals according to IBP strata. It is noteworthy that deaths without address information on the SIM were excluded from the analysis due to the impossibility of identifying the census tract and, consequently, classifying them in one of the IBP categories. The assessment of inequality in the distribution of mortality was carried out by comparative analysis of the rates and the percentage variation between the IBP strata (low, medium, and high deprivation).

### Ethical aspects

This research complies with Resolution No. 466, of December 12^th^, 2012, of the National Health Council (*Conselho Nacional de Saúde* – CNS). It was approved by the Research Ethics Committee of UFMG under the reference number 3.258.076.

## RESULTS

Overall, it was not possible to geocode about 30% of the deaths. This loss was greater in the first three years in some capitals in the Northeast, such as Fortaleza, João Pessoa, and Salvador. In the second triennium, the percentage of losses was lower, an average of 28% (Figure S1 supplementary material).

Considering the set of capitals in the three-year period 2010 to 2012, the mortality rates due to NCDs before GC redistribution were equivalent to 202.9 in the low deprivation stratum of the IBP, 258.4 in the medium deprivation stratum, and 249.6 per 100,000 inhabitants in the high deprivation stratum. After the adjustments and redistribution of GC, the values of the rates increased to 220.5, 285.5, and 279.4 per 100,000 inhabitants in the low, medium, and high deprivation strata, in that order (Table 1). There was, therefore, an increase of 8.7, 10.5, and 11.9%, respectively (Table S1 supplementary material).

**Table 1:** Age-standardized premature mortality rates from noncommunicable chronic diseases, per 100,000 inhabitants, before and after redistribution of garbage causes, according to categories of the Brazilian Deprivation Index. Capitals of Brazil, 2010 to 2012.

Regions	Capitals	Premature mortality rate from NCDs					
		No GC redistribution			With GC redistribution		
		Low	Medium	High	Low	Medium	High
**Total**		**202.9**	**258.4**	**249.6**	**220.5**	**285.5**	**279.4**
Center-West	Distrito Federal	135.2	217.1	179.7	143.4	232.9	194.3
	Campo Grande	224.9	280.7	309.9	235.6	291.9	324.3
	Cuiabá	214.9	248.9	262.9	227.8	266.9	286.1
	Goiânia	166.2	222.9	259.5	182.3	243.6	284.9
Northeast	Aracaju	145.6	203.8	240.9	159.6	222.9	271.3
	Fortaleza	97.5	120.4	126.5	105.0	133.4	142.8
	João Pessoa	128.7	186.0	184.2	138.0	200.7	206.8
	Maceió	145.9	185.4	239.9	155.8	201.3	264.4
	Natal	150.8	234.0	259.7	159.7	243.3	276.4
	Recife	178.3	244.7	322.2	185.9	257.1	341.8
	Salvador	115.5	181.2	220.9	127.1	211.2	260.9
	São Luís	156.3	158.6	219.6	165.1	170.5	232.7
	Teresina	134.7	180.0	205.5	144.9	190.9	223.9
North	Belém	154.6	193.5	209.2	171.9	219.4	244.9
	Boa Vista	164.7	222.9	227.3	189.0	258.5	253.9
	Macapá	212.4	150.6	149.1	238.5	175.3	183.4
	Manaus	171.7	223.4	239.6	192.7	261.2	284.7
	Palmas	173.7	199.1	226.2	182.9	208.5	237.7
	Porto Velho	183.8	205.6	216.9	203.6	231.9	249.9
	Rio Branco	155.1	162.2	220.2	168.7	175.2	252.1
Southeast	Belo Horizonte	163.2	233.1	244.9	184.8	270.9	287.4
	Rio de Janeiro	234.1	304.3	297.6	266.6	356.3	353.0
	São Paulo	233.5	300.1	306.0	248.8	324.2	334.1
	Vitória	145.4	311.8	291.7	151.5	332.8	314.4
South	Curitiba	229.1	299.6	329.5	238.4	313.3	345.8
	Florianópolis	187.9	222.6	264.9	197.8	233.6	291.7
	Porto Alegre	239.9	331.5	281.3	252.5	353.6	303.9

NCDs: noncommunicable chronic diseases; GC: garbage causes.

This pattern of higher rates after GC redistribution was observed in all capitals in the first three years and, in general, rates were higher also in the highest deprivation stratum. After redistribution of GC, the increase in rates in intra-urban areas with low deprivation ranged from 4.1% (Curitiba) to 14.8% (Boa Vista); in areas with medium deprivation, it varied from 4.0% (Natal) to 17.1% (Rio de Janeiro) and, in the high deprivation stratum, it varied from 4.6% (Campo Grande) to 23.0% (Macapá) (Table S1 supplementary material).

Still in the first three years, the rates after redistribution of GC in the low deprivation stratum ranged from 105.0 in Fortaleza to 266.6 per 100,000 inhabitants in Rio de Janeiro. In the medium deprivation stratum, Fortaleza had the lowest rate, of 133.4, and Rio de Janeiro the highest, of 356.3 per 100 thousand inhabitants. In the high deprivation stratum, the lowest rate occurred in Fortaleza and the highest rate in Rio de Janeiro, respectively, 142.8 and 353 per 100,000 inhabitants (Table 1 and Table S1 supplementary material).

In the three-year period 2017 to 2019, mortality rates before GC redistribution corresponded to 163.9 in the low deprivation stratum, 224.5 in medium deprivation, and 231.9 per 100,000 inhabitants in the high deprivation stratum (Table 2). Rates increased after GC redistribution, rising to 180.3, 248.7, and 258.4 per 100,000 inhabitants in these strata, an increase of 10, 10.8, and 11.4%, respectively (Table 2 and Table S2 supplementary material).

**Table 2:** Age-standardized premature mortality rates from noncommunicable chronic diseases, per 100,000 inhabitants, before and after redistribution of garbage causes, according to categories of the Brazilian Deprivation Index. Capitals of Brazil, 2017 to 2019.

Regions	Capitals	Total NCDs					
		No GC redistribution			With GC redistribution		
		Low	Medium	High	Low	Medium	High
**Total**		**163.9**	**224.5**	**231.9**	**180.3**	**248.7**	**258.4**
Center-West	Distrito Federal	97.4	175.6	160.5	101.9	184.7	170.3
	Campo Grande	180.2	244.7	305.4	190.1	257.7	321.3
	Cuiabá	178.4	228.2	268.3	186.7	235.8	280.2
	Goiânia	154.2	199.7	261.6	160.1	208.9	272.9
Northeast	Aracaju	127.4	160.8	207.4	138.5	177.9	232.8
	Fortaleza	95.1	133.3	139.4	103.7	145.9	152.4
	João Pessoa	122.1	144.8	152.4	130.1	158.2	170.6
	Maceió	131.2	156.9	216.9	139.8	168.9	236.7
	Natal	142.3	220.4	255.1	148.9	234.6	274.7
	Recife	151.1	225.1	307.6	156.6	234.4	322.7
	Salvador	105.0	164.5	202.8	118.2	188.9	240.5
	São Luís	98.9	123.2	164.1	109.7	132.4	178.1
	Teresina	130.9	144.6	185.2	138.9	155.5	197.7
North	Belém	128.3	177.9	192.6	140.1	191.6	210.8
	Boa Vista	193.9	258.9	297.7	205.2	272.5	311.2
	Macapá	184.1	205.9	198.8	218.8	244.1	234.9
	Manaus	135.1	171.0	197.4	157.2	203.1	238.0
	Palmas	128.3	127.8	153.6	132.2	132.9	158.3
	Porto Velho	145.3	179.8	210.6	166.5	208.9	241.9
	Rio Branco	180.2	179.8	226.8	213.8	199.1	261.8
Southeast	Belo Horizonte	128.5	207.7	241.9	143.5	233.7	272.2
	Rio de Janeiro	213.2	295.5	293.3	243.8	345.9	348.2
	São Paulo	183.4	254.2	281.2	200.3	280.2	310.6
	Vitória	124.9	285.6	273.4	128.9	295.0	279.4
South	Curitiba	141.9	176.9	189.6	149.9	186.4	203.9
	Florianópolis	166.4	252.9	273.4	175.9	267.3	290.5
	Porto Alegre	186.5	294.7	249.4	205.0	318.1	271.6

NCDs: noncommunicable chronic diseases; GC: garbage causes.

In general, rates increased in capitals after GC redistribution and were higher in high deprivation strata. The percentage variation was lower in Vitória, compared to the other capitals, in the three deprivation strata: 3.2, 3.3, and 2.2% in the census tracts of low, medium, and high deprivation, respectively. The variation was higher in Macapá in the same strata: 18.8, 18.6, and 18.2% (Table S2 supplementary material).

In the low deprivation stratum, mortality rates after GC redistribution ranged from 101.9 in the Federal District to 243.8 per 100,000 inhabitants in Rio de Janeiro. In the medium deprivation stratum, rates ranged from 132.4 in São Luís to 345.9 per 100,000 inhabitants in Rio de Janeiro; as for the high deprivation stratum, the lowest rate was in Fortaleza and the highest in Rio de Janeiro: 152.4 and 348.2 per 100,000 inhabitants, respectively (Table 2 and Table S2 supplementary material).

Analyzing the evolution of adjusted rates over the two triennia, a reduction was observed in the capitals as a whole, in all deprivation strata. The greatest decrease occurred in the low deprivation stratum (-18.2%) and the smallest in the high deprivation stratum (-7.5%) (Table 3). The capitals with the greatest reduction in the low-deprivation stratum were: Curitiba (-37.1%), São Luís (-33.6%), Federal District (-28.9%), Palmas (-27.7%), and Belo Horizonte (-22.3%). In the medium deprivation stratum, the greatest decreases occurred in Curitiba (-40.5%), and Palmas (-36.3%), and in the high deprivation stratum the most significant decreases occurred in Curitiba (-41.0%), Palmas (-33.4%), and São Luís (-23.5%). Two capitals showed an increase in rates in the three deprivation strata: Rio Branco (26.7, 13.6, 3.8%) and Boa Vista (8.6, 5.4, and 22.6%). Macapá (39.2 and 28.1%) and Fortaleza (9.4 and 6.7%) had an increase in the medium and high deprivation strata (Table 3).

**Table 3:** Percentage variation in premature mortality from noncommunicable chronic diseases between the 2010-2012 and 2017-2019 trienniums, according to the Brazilian Deprivation Index, after the redistribution of garbage causes in the capitals.

Regions	Capitals	Percentage variation (%) IBP		
		Lo	Me	Hi
**Total**		**−18.2**	**−12.9**	**−7.5**
Center-West	Distrito Federal	−28.9	−20.7	−12.4
	Campo Grande	−19.3	−11.7	−0.9
	Cuiabá	−18.0	−11.7	−2.1
	Goiânia	−12.2	−14.2	−4.2
Northeast	Aracaju	−13.2	−20.2	−14.2
	Fortaleza	−1.2	9.4	6.7
	João Pessoa	−5.7	−21.2	−17.5
	Maceió	−10.3	−16.1	−10.5
	Natal	−6.8	−3.6	−0.6
	Recife	−15.8	−8.8	−5.6
	Salvador	−7.0	−10.6	−7.8
	São Luís	−33.6	−22.3	−23.5
	Teresina	−4.1	−18.5	−11.7
North	Belém	−18.5	−12.7	−13.9
	Boa Vista	8.6	5.4	22.6
	Macapá	−8.3	39.2	28.1
	Manaus	−18.4	−22.2	−16.4
	Palmas	−27.7	−36.3	−33.4
	Porto Velho	−18.2	−9.9	−3.2
	Rio Branco	26.7	13.6	3.8
Southeast	Belo Horizonte	−22.3	−13.7	−5.3
	Rio de Janeiro	−8.6	−2.9	−1.4
	São Paulo	−19.5	−13.6	−7.0
	Vitória	−14.9	−11.4	−11.1
South	Curitiba	−37.1	−40.5	−41.0
	Florianópolis	−11.1	14.4	−0.4
	Porto Alegre	−18.8	−10.0	−10.6

IBP: Brazilian Deprivation Index (*Índice Brasileiro de Privação*); Lo: low deprivation; Me: medium deprivation; Hi: high deprivation; NCDs: noncommunicable chronic diseases.

## DISCUSSION

The study showed the impact of correcting death data in estimating premature mortality rates due to NCDs. It was observed that rates increased between 8 and 12% among deprivation strata after GC redistribution, and the greatest increases occurred in the most vulnerable areas. When considering the initial and final three-year periods, there were significant decreases in mortality in most Brazilian capitals, with the most significant decreases in low vulnerability strata. Between the triennia, reductions above 20% in all deprivation strata were observed in Curitiba, São Luís, and Palmas. On the other hand, increases in the rates were observed in the last three years in the capitals Rio Branco, Boa Vista, Macapá, and Fortaleza.

SIM was created to regularly obtain data on mortality in the country and has universal coverage. Advances related to expanding the coverage of deaths, data completeness, and better definition of the underlying causes of death, through the reduction in the proportion of GC^2^, prove the good results of actions by the Ministry of Health to qualify mortality data in the country. However, the treatment of these data proves to be important, especially in space-time comparative analyses, since the quality of this information is heterogeneous in the country, in which the North and, above all, the Northeast regions still represent the worst scenarios^26^.

In this sense, the use of raw data requires attention, and it is recommended the implementation of correction methods to the SIM data before carrying out analyses of the health situation, especially at the subnational level^16,23^. Correction of underreported deaths is recommended, as well as consideration of ill-defined causes and GC through redistribution methods^16,23^. The present study reinforces the importance of adopting these methods when assessing the impact on rates before and after processing death data in small areas.

The study innovates by presenting corrected data with a methodology that considers GC redistribution. The methodology proposed by Teixeira et al.^22^ introduces specific GC redistribution algorithms for Brazil, since it considers the results of death investigations initiated in the project with 60 cities^2^. By proposing this empirical methodology based on investigations carried out in state health departments, the methodology considers the Brazilian context, which differs from that observed in other countries^23,27^.

Analyzing the estimates after processing the data, an important finding was the reduction in mortality rates due to NCDs in most Brazilian capitals, especially in the less vulnerable strata. Malta *et al*.^5^, when analyzing GBD data between 1990 and 2017, identified a 35.9% decline in premature mortality due to NCDs, with cardiovascular diseases having the most significant reduction, of 47.9%. These advances can be explained by: the improvement in living and health conditions; poverty reduction; greater access to goods and services; and expansion of the Brazilian Health System (*Sistema Único de Saúde* – SUS) with increased coverage of its list of services, as well as primary care, which made it possible to meet the demands of the Brazilian population driven by epidemiological, nutritional, and demographic transitions^28^. Added to this are the advances in health policies, including the expansion of the Family Health Program; implementation of the Strategic Action Plan to Tackle NCDs^7,13^; the National Health Promotion Policy^29^; and implementation of regulatory measures, such as the law on tobacco-free environments^30^, among others.

On the other hand, some capitals in the North and Northeast showed an increase in rates between the triennia, which can be explained by the improvement in the quality of mortality information in recent decades and advances related to the expansion of death coverage, more accurate notification of the causes of death and the decrease in the proportion of GC^2,27^.

Differences in the rates among IBP categories, with higher rates in most capitals and in both three-year periods in the most deprived strata, represent an important result with regards to the assessment of health inequalities at the intra-urban scale. These differences have been well documented in the literature, which suggests that the low-income population with less education has higher rates of NCDs^8^ and its risk factors^31,32^ due to social inequalities and low access to health goods and services^8^. In this sense, an analysis of the 2013 Brazilian National Health Service (*Pesquisa Nacional de Saúde* – PNS) on the prevalence of health behaviors in the Brazilian population revealed profound social inequalities: the less educated, brown or black and those without private health insurance had a higher prevalence of smoking, physical inactivity during leisure time, sedentary lifestyle, consumption of whole milk, and low consumption of greens, vegetables, and fruits^31^. In addition, black or brown individuals had a higher prevalence of excessive alcohol consumption compared to white ones^32^.

The increase in the burden of NCDs poses challenges for Brazilian public health. In addition to unequally affecting populations, by having a more negative impact on those in a more vulnerable situation from a social and economic point of view, NCDs represent an obstacle to socioeconomic development^12^. Despite advances in tackling NCDs, the goal of reducing mortality due to these diseases by 30% by 2030 should not be achieved^5^. To this end, it is necessary to move forward with coordinated action between the federal, state, and municipal governments, aimed at expanding and improving the capacity to offer and promote access to health services; to adopt or improve regulatory measures to control unhealthy foods, tobacco, alcohol, among other risk factors; and preventive and treatment measures^3,5,14^.

The study has some limitations. Among them, the use of the IBP, which was constructed with data from the 2010 census and may not capture changes in the distribution of deprivation over the last decade. This can only be measured after the 2022 demographic census. In addition, considering the capitals as a whole, 30% of deaths, on average, could not be geocoded and, therefore, were not classified in the IBP categories. It is noteworthy that mortality rates may be even higher in areas of greater deprivation, due to the higher proportion of non-georeferenced deaths in these areas. The analyses were limited to capital cities due to the better quality of death information in these locations. This reflects in the production of more accurate estimates. As a strength, the study innovates in methodological advances, such as the treatment of missing data and the redistribution of GC, contributing to the body of knowledge on the improvement of mortality estimates in the country. Furthermore, for the first time, the IBP was used to calculate inequalities in estimates of mortality due to NCDs in the Brazilian capitals.

In short, the present study showed that the redistribution of GC represented an increase in the values of mortality rates due to NCDs in the investigated municipalities, and the increase was greater in the strata of greater social deprivation. Higher mortality rates were observed in strata with greater social deprivation. In the analysis of the temporal evolution, there was a decrease in premature mortality due to NCDs, especially in areas of lesser social deprivation.

The use of methodology for processing mortality data is constantly being developed, however it is important to encourage the improvement of surveillance and the completion of death certificates, and to reinforce training and continuing education of professional figures delivering death notifications.
