# Supplementary material for: Premature mortality due to noncommunicable diseases in Brazilian capitals: redistribution of garbage causes and evolution by social deprivation strata
Source: Rev Bras Epidemiol. 2023 Apr 21;26(Suppl 1):e230002. doi: 10.1590/1980-549720230002.supl.1 (PMC10184710; doi:10.1590/1980-549720230002.supl.1)
Supplement: Supplementary file 1 [file 1980-5497-rbepid-26-suppl1-e230002-suppl1.pdf]

Figura S1 – Percentual de óbitos não geocodificados. Sistema de Informação de Mortalidade, capitais do Brasil, triênios 2010 a 2012 e 2017 a 2019.

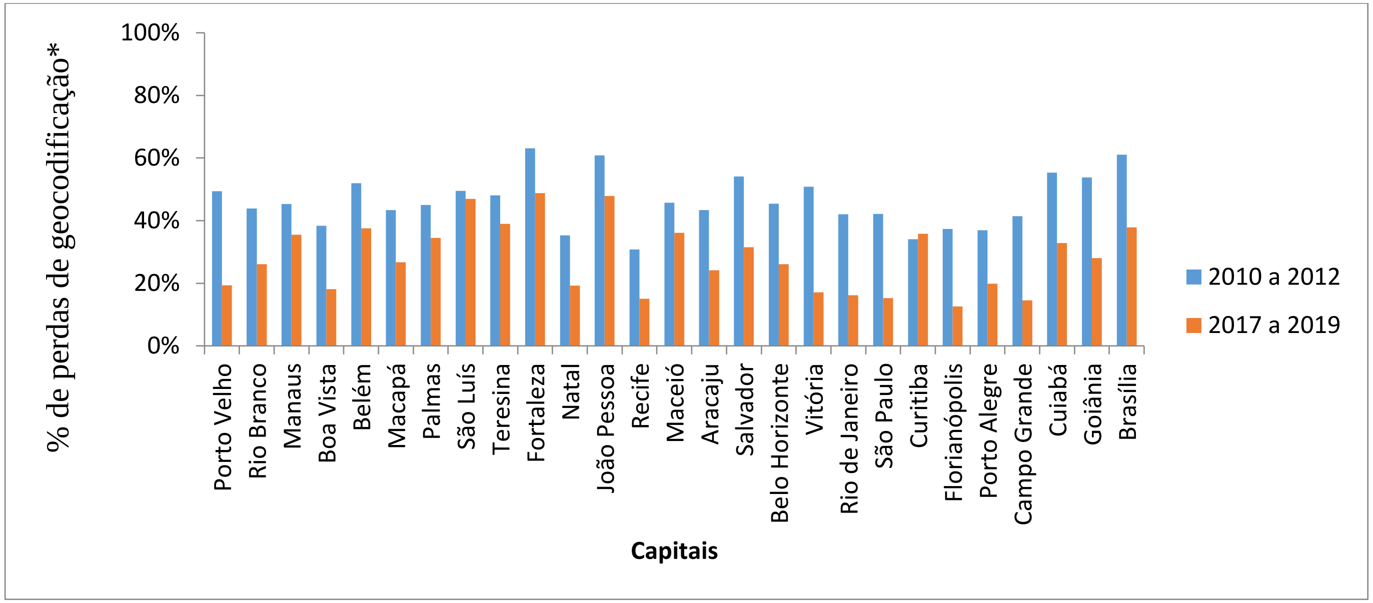

*Óbitos sem informação do setor censitário correspondente ao endereço de residência do indivíduo.

Tabela S1 - Taxas de mortalidade prematura por doenças crônicas não transmissíveis, por 100 mil habitantes, antes e após a redistribuição de causas *garbage* (CG), intervalos de confiança de 95% e variação percentual entre as categorias do Índice Brasileiro de Privação (IBP). Capitais do Brasil, 2010 a 2012.

Regiões	Capitais	Taxa de mortalidade por DCNT (IC95%)						Variação Percentual (%)		
		Sem redistribuição de CG			Com redistribuição de CG					
		Ba	Me	A	Ba	Me	A	Ba	Me	A
	Total	**202,9(201,2-204,6)**	**258,4(255,9-260,9)**	**249,6(247,5-251,8)**	***220,5(218,7-222,3)***	***285,5(282,8-288,1)***	***279,4(277,1-281,7)***	***8,7***	***10,5***	***11,9***
**Centro-Oeste**	Distrito Federal	135,2(129,2-141,1)	217,1(207,6-226,6)	179,7(168,9-190,5)	143,4(137,3-149,5)	232,9(223,2-242,8)	194,3(183,1-205,5)	6,1	7,3	8,1
	Campo Grande	224,9(208,8-241,2)	280,7(262-299,4)	309,9(293,2-326,9)	235,6(219-252,2)	291,9(272,9-311,1)	324,3(307,1-341,6)	4,8	4,0	4,6
	Cuiabá	214,9(196,9-233)	248,9(227,9-270,1)	262,9(240,8-285,1)	227,8(209,2-246,4)	266,9(245,1-288,8)	286,1(263-309,2)	6,0	7,2	8,8
	Goiânia	166,2(157,4-175)	222,9(209,7-236,4)	259,5(244,2-274,7)	182,3(173,1-191,6)	243,6(229,7-257,5)	284,9(268,9-300,9)	9,7	9,3	9,8
**Nordeste**	Aracaju	145,6(130,3-160,9)	203,8(182,7-225)	240,9(223,4-258,4)	159,6(143,6-175,7)	222,9(200,8-245)	271,3(252,7-289,8)	9,6	9,4	12,6
	Fortaleza	97,5(89,9-105)	120,4(111,9-128,9)	126,5(121,3-131,8)	105(97,1-112,8)	133,4(124,4-142,4)	142,8(137,2-148,3)	7,7	10,8	12,9
	João Pessoa	128,7(115,2-142,2)	186(168,9-203,1)	184,2(171,6-196,8)	138(124-151,9)	200,7(183-218,5)	206,8(193,5-220,2)	7,2	7,9	12,3
	Maceió	145,9(127,3-164,7)	185,4(162,7-208,1)	239,9(229,3-250,4)	155,8(136,5-175,1)	201,3(177,6-224,9)	264,4(253,3-275,5)	6,8	8,6	10,2
	Natal	150,8(135,6-166,1)	234(213,1-254,9)	259,7(246,8-272,6)	159,7(144-175,4)	243,3(222-264,6)	276,4(263,1-289,8)	5,9	4,0	6,4
	Recife	178,3(166,9-189,7)	244,7(228,8-260,5)	322,2(312,5-331,9)	185,9(174,2-197,5)	257,1(240,8-273,3)	341,8(331,8-351,8)	4,3	5,1	6,1
	Salvador	115,5(108,7-122,3)	181,2(172,3-190,1)	220,9(213,7-228,2)	127,1(120-134,2)	211,2(201,7-220,8)	260,9(253,1-268,8)	10,0	16,6	18,1
	São Luís	156,3(139,7-173)	158,6(142,9-174,3)	219,6(208,1-231,1)	165,1(148-182,3)	170,5(154,2-186,8)	232,7(220,9-244,6)	5,6	7,5	6,0
	Teresina	134,7(116,3-153)	180(159,5-200,6)	205,5(194,3-216,6)	144,9(125,8-163,9)	190,9(169,7-212)	223,9(212,3-235,6)	7,6	6,1	9,0
**Norte**	Belém	154,6(141,6-167,6)	193,5(181-205,9)	209,2(199,8-218,7)	171,9(158,2-185,6)	219,4(206,1-232,6)	244,9(234,7-255,1)	11,2	13,4	17,1
	Boa Vista	164,7(122-207,3)	222,9(179,5-266,4)	227,3(203,7-250,9)	189(143,3-234,7)	258,5(211,6-305,4)	253,9(229-278,9)	14,8	16,0	11,7
	Macapá	212,4(148,8-276)	150,6(111,3-189,9)	149,1(134,4-163,7)	238,5(171,1-305,9)	175,3(133-217,7)	183,4(167,1-199,8)	12,3	16,4	23,0
	Manaus	171,7(154,9-188,5)	223,4(208,9-238)	239,6(230,3-249)	192,7(174,9-210,5)	261,2(245,4-277)	284,7(274,5-294,9)	12,2	16,9	18,8
	Palmas	173,7(139-208,3)	199,1(154,1-244,1)	226,2(194,6-257,9)	182,9(147,4-218,4)	208,5(162,6-254,5)	237,7(205,3-270,1)	5,3	4,7	5,1
	Porto Velho	183,8(145,9-221,8)	205,6(175,4-235,7)	216,9(199,6-234,2)	203,6(163,6-243,5)	231,9(199,8-264)	249,9(231,3-268,5)	10,8	12,8	15,2
	Rio Branco	155,1(115,1-195)	162,2(128,7-195,7)	220,2(200,9-239,6)	168,7(127-210,4)	175,2(140,4-210)	252,1(231,4-272,8)	8,8	8,0	14,5
**Sudeste**	Belo Horizonte	163,2(158-168,4)	233,1(222,9-243,4)	244,9(230,8-259,1)	184,8(179,3-190,3)	270,9(259,9-282,1)	287,4(272,1-302,7)	13,2	16,2	17,3
	Rio de Janeiro	234,1(230,1-238,1)	304,3(297,5-311,1)	297,6(290,4-304,7)	266,6(262,3-270,9)	356,3(349-363,6)	353(345,2-360,8)	13,9	17,1	18,6
	São Paulo	233,5(230,1-237)	300,1(295,3-304,9)	306(300,6-311,4)	248,8(245,2-252,4)	324,2(319,3-329,2)	334,1(328,4-339,7)	6,6	8,0	9,2
	Vitória	145,4(131,7-159,1)	311,8(280,6-343,1)	291,7(254,7-328,7)	151,5(137,6-165,5)	332,8(300,6-365,1)	314,4(275,9-352,8)	4,2	6,7	7,8
**Sul**	Curitiba	229,1(222,4-235,8)	299,6(284,5-314,7)	329,5(301,1-357,9)	238,4(231,6-245,3)	313,3(297,8-328,8)	345,8(316,7-374,9)	4,1	4,6	4,9
	Florianópolis	187,9(174,8-201,1)	222,6(199,1-246)	264,9(227,1-302,7)	197,8(184,3-211,3)	233,6(209,7-257,6)	291,7(252,1-331,2)	5,3	4,9	10,1
	Porto Alegre	239,9(232,4-247,4)	331,5(313,9-349,1)	281,3(263,2-299,4)	252,5(244,7-260,2)	353,6(335,4-371,8)	303,9(285,2-322,7)	5,3	6,7	8,0

Legenda: Ba = baixa privação; Me = média privação, A = alta privação. DCNT = doenças crônicas não transmissíveis. CG = causas *garbage*.

Tabela S2 - Taxas de mortalidade prematura por doenças crônicas não transmissíveis, por 100 mil habitantes, antes e após a redistribuição de causas *garbage* (CG), intervalos de confiança de 95% e variação percentual entre as categorias do Índice Brasileiro de Privação (IBP). Capitais do Brasil, 2017 a 2019.

Regiões	Capitais	Taxa de mortalidade por DCNT (IC95%)						Variação Percentual (%)		
		Sem redistribuição de CG			Com redistribuição de CG					
		**Ba**	**Me**	**A**	**Ba**	**Me**	**A**	**Ba**	**Me**	**A**
	Total	**163,9(162,5-165,3)**	**224,5(222,3-226,6)**	**231,9(230,1-233,8)**	**180,3(178,8-181,7)**	**248,7(246,5-250,9)**	**258,4(256,4-260,4)**	**10,0**	**10,8**	**11,4**
**Centro-Oeste**	Distrito Federal	97,4(93,1-101,7)	175,6(168,3-182,8)	160,5(151,9-169,2)	101,9(97,6-106,4)	184,7(177,3-192,1)	170,3(161,4-179,2)	4,6	5,2	6,1
	Campo Grande	180,2(167,3-193)	244,7(229,1-260,2)	305,4(290,6-320,2)	190,1(176,9-203,4)	257,7(241,8-273,6)	321,3(306,2-336,5)	5,5	5,3	5,2
	Cuiabá	178,4(163,9-192,8)	228,2(210,3-246,2)	268,3(248,4-288,2)	186,7(171,9-201,6)	235,8(217,5-254)	280,2(259,8-300,5)	4,7	3,3	4,4
	Goiânia	154,2(146,6-161,7)	199,7(188,6-210,8)	261,6(248,3-275)	160,1(153,3-168,7)	208,9(197,7-220,4)	272,9(259,3-286,7)	3,8	4,6	4,3
**Nordeste**	Aracaju	127,4(114,9-139,8)	160,8(144,3-177,2)	207,4(193,2-221,6)	138,5(125,5-151,4)	177,9(160,6-195,1)	232,8(217,8-247,9)	8,7	10,6	12,2
	Fortaleza	95,1(88,4-101,9)	133,3(125,2-141,4)	139,4(134,5-144,3)	103,7(96,6-110,7)	145,9(137,5-154,4)	152,4(147,3-157,6)	9,0	9,5	9,3
	João Pessoa	122,1(110,5-133,7)	144,8(131,5-158,1)	152,4(142,3-162,5)	130,1(118,9-143,1)	158,2(144,3-172,1)	170,6(160-181,3)	6,6	9,3	11,9
	Maceió	131,2(115,7-146,7)	156,9(138,6-175,1)	216,9(208,2-225,8)	139,8(123,8-155,8)	168,9(150-188)	236,7(227,5-245,9)	6,6	7,6	9,1
	Natal	142,3(129,1-155,6)	220,4(202,1-238,7)	255,1(243,6-266,6)	148,9(135,4-162,6)	234,6(215,7-253,5)	274,7(262,8-286,7)	4,6	6,4	7,7
	Recife	151,1(141,5-160,6)	225,1(211,1-239)	307,6(299-316,3)	156,6(146,9-166,4)	234,4(220,2-248,6)	322,7(313,8-331,5)	3,6	4,1	4,9
	Salvador	105(99,3-110,7)	164,5(157-172)	202,8(196,7-208,9)	118,2(112,1-124,3)	188,9(181-197,1)	240,5(233,9-247,2)	12,6	14,8	18,6
	São Luís	98,9(87,2-110,7)	123,2(110,9-135,5)	164,1(155,3-172,9)	109,7(97,3-122,2)	132,4(119,7-145,2)	178,1(168,9-187,3)	10,9	7,5	8,5
	Teresina	130,9(114,8-147)	144,6(128,2-160,9)	185,2(175,9-194,5)	138,9(122,3-155,5)	155,5(138,5-172,4)	197,7(188,1-207,4)	6,1	7,5	6,7
**Norte**	Belém	128,3(117,4-139,1)	177,9(167-188,8)	192,6(184,3-200,9)	140,1(128,7-151,5)	191,6(180,3-202,9)	210,8(202,2-219,5)	9,2	7,7	9,4
	Boa Vista	193,9(156,7-231,3)	258,9(221,3-296,7)	297,7(275,9-319,4)	205,2(166,8-243,5)	272,5(233,8-311,1)	311,2(289-333,5)	5,8	5,3	4,5
	Macapá	184,1(135-233,2)	205,9(168,1-243,8)	198,8(184,7-212,9)	218,8(165,3-272,4)	244,1(202,9-285,3)	234,9(219,6-250,1)	18,8	18,6	18,2
	Manaus	135,1(122,5-147,7)	171(160,2-181,8)	197,4(190,3-204,6)	157,2(143,6-170,8)	203,1(191,4-214,9)	238(230,1-245,8)	16,4	18,8	20,6
	Palmas	128,3(105,3-151,2)	127,8(100,5-155)	153,6(133,5-173,6)	132,2(108,9-155,5)	132,9(105,1-160,7)	158,3(138-178,6)	3,0	4,0	3,1
	Porto Velho	145,3(117,1-173,4)	179,8(156,3-203,3)	210,6(196,6-224,6)	166,5(136,4-196,7)	208,9(183,5-234,2)	241,9(226,8-256,9)	14,6	16,2	14,9
	Rio Branco	180,2(143,5-217)	179,8(149,4-210,2)	226,8(210-243,6)	213,8(173,7-253,8)	199,1(167,1-231,1)	261,8(243,7-280)	18,6	10,7	15,4
**Sudeste**	Belo Horizonte	128,5(124,2-132,8)	207,7(198,7-216,6)	241,9(229-254,9)	143,5(139-148)	233,7(224,2-243,2)	272,2(258,5-285,9)	11,7	12,5	12,5
	Rio de Janeiro	213,2(209,6-216,9)	295,5(289,1-301,8)	293,3(286,6-300)	243,8(239,9-247,7)	345,9(339,1-352,8)	348,2(340,9-355,5)	14,4	17,1	18,7
	São Paulo	183,4(180,6-186,2)	254,2(250,2-258,3)	281,2(276,5-286)	200,3(197,4-203,3)	280,2(276-284,4)	310,6(305,6-315,6)	9,2	10,2	10,5
	Vitória	124,9(113,6-136,2)	285,6(258,7-312,5)	273,4(241-305,8)	128,9(117,4-140,4)	295(267,6-322,4)	279,4(246,7-312,2)	3,2	3,3	2,2
**Sul**	Curitiba	141,9(137,3-146,8)	176,9(166,5-187,5)	189,6(170,2-209)	149,9(145-154,8)	186,4(175,7-197,2)	203,9(183,8-224)	5,6	5,4	7,5
	Florianópolis	166,4(155,6-177,2)	252,9(231,4-274,6)	273,4(239,9-306,9)	175,9(164,8-187,1)	267,3(245-289,5)	290,5(256-325)	5,7	5,7	6,3
	Porto Alegre	186,5(180,2-192,8)	294,7(279,1-310,2)	249,4(233,4-265,4)	205(198,4-211,6)	318,1(301,9-334,3)	271,6(254,9-288,3)	9,9	7,9	8,9

Legenda: Ba = baixa privação; Me = média privação, AS = alta privação. DCNT = doenças crônicas não transmissíveis. CG = causas *garbage*.
