# Supplementary material for: Premature mortality due to noncommunicable diseases in Brazilian capitals: redistribution of garbage causes and evolution by social deprivation strata
Source: Rev Bras Epidemiol. 2023 Apr 21;26(Suppl 1):e230002. doi: 10.1590/1980-549720230002.supl.1 (PMC10184710; doi:10.1590/1980-549720230002.supl.1)
Supplement: Translation

# Translation: Mortalidade prematura por doenças crônicas não transmissíveis em capitais brasileiras: redistribuição de causas garbage e evolução por estratos de privação social

**DOI:** 10.1590/1980-549720230002.supl.1.1

**Authors:** Deborah Carvalho Malta, Renato Azeredo Teixeira, Laís Santos de Magalhães Cardoso, Juliana Bottoni de Souza, Regina Tomie Ivata Bernal, Pedro Cisalpino Pinheiro, Crizian Saar Gomes, Alastair Leyland, Ruth Dundas, Maurício Lima Barreto

**Affiliations:** I Universidade Federal de Minas Gerais, Escola de Enfermagem, Departamento de Enfermagem Materno Infantil e Saúde Pública – Belo Horizonte (MG), Brasil.; II Universidade Federal de Minas Gerais, Faculdade de Medicina, Programa de Pós-Graduação em Saúde Pública – Belo Horizonte (MG), Brasil.; III Universidade Federal de Minas Gerais, Escola de Enfermagem, Programa de Pós-Graduação em Enfermagem – Belo Horizonte (MG), Brasil.; IV Universidade Federal de Minas Gerais, Escola de Enfermagem, Observatório de Doenças e Agravos Não Transmissíveis – Belo Horizonte (MG), Brasil.; V University of Glasgow, MRC/CSO Social & Public Health Sciences Unit – Glasgow, Reino Unido.; VI Fundação Oswaldo Cruz, Centro de Integração de Dados e Conhecimentos para Saúde – Salvador (BA), Brasil.; VII Universidade Federal da Bahia, Instituto de Saúde Coletiva – Salvador (BA), Brasil.

**Keywords:** Doenças não transmissíveis, Mortalidade prematura, Registros de mortalidade, Disparidades nos níveis de saúde, Análise de pequenas áreas

## Abstract

**Objetivo:**

Analisar a mortalidade prematura por doenças crônicas não transmissíveis (DCNT) nas capitais brasileiras e Distrito Federal (DF) após redistribuição das causas *garbage*, e a evolução temporal segundo estratos de privação social nos triênios 2010 a 2012 e 2017 a 2019.

**Métodos:**

Foram aplicadas correções ao Sistema de Informação sobre Mortalidade (SIM), sendo empregada metodologia para redistribuição das causas *garbage* (CG). As taxas de mortalidade prematura por DCNT padronizadas por idade foram estimadas. Foram analisadas as diferenças entre as taxas de mortalidade por DCNT segundo categorias do Índice Brasileiro de Privação (IBP) e entre os triênios.

**Resultados:**

No conjunto das capitais, as taxas aumentaram entre 8 e 12% após a redistribuição de CG, e os maiores acréscimos ocorreram em áreas de alta privação: 11,9 e 11,4%, triênios 1 e 2, respectivamente. Houve variabilidade entre as capitais. Observou-se redução das taxas em todos os estratos de privação entre os triênios, sendo maior decréscimo no estrato de baixa privação (-18,2%), e menor no estrato de alta privação (-7,5%).

**Conclusão:**

A redistribuição de CG representou aumento das taxas de mortalidade, sendo maior nos estratos de maior privação social. Via de regra, observou-se gradiente positivo de mortalidade com o aumento da privação social. A análise da evolução temporal evidenciou decréscimo da mortalidade por DCNT entre os triênios, sobretudo em áreas de menor privação social.

## INTRODUÇÃO

As Doenças Crônicas Não Transmissíveis (DCNT) representam a maior causa de morbimortalidade no Brasil e no mundo^1,2^. Estima-se que sejam responsáveis por 41 milhões de mortes no mundo por ano e, desse total, 15 milhões são consideradas prematuras por acometerem indivíduos com idades entre 30 e 69 anos^3,4^. No Brasil, em 2019, a mortalidade geral por DCNT correspondeu a cerca de 76% do total de óbitos, sendo que 66,1% foram mortes prematuras^5^.

As DCNT impõem uma carga significativa e crescente sobre os sistemas de saúde e a economia geral dos países^6^. Estima-se que o crescimento econômico seja reduzido em até 0,5% para cada incremento de 10% na mortalidade por DCNT. Assim, as DCNT se destacam entre as principais ameaças globais ao desenvolvimento econômico, bem como à saúde e ao bem-estar das populações, assim, monitorá-las se torna essencial^7^.

Os determinantes sociais contribuem fortemente no aumento e na gravidade das DCNT^8^. Essas doenças e respectivos fatores de risco impactam mais, de forma negativa, as populações socioeconomicamente vulneráveis, uma vez que estas possuem menor acesso aos serviços de saúde e a ambientes considerados saudáveis^9^. Esse déficit, por sua vez, reduz as oportunidades de prevenção e promoção da saúde entre essas populações^10–12^.

Diversos compromissos nacionais e globais foram estabelecidos visando à redução das DCNT, entre eles o Plano de Ações Estratégicas para o Enfrentamento das DCNT no Brasil 2011–2022^13^, o Plano Global da Organização Mundial da Saúde (OMS) de enfrentamento das DCNT^14^ e a Agenda 2030 da Organização das Nações Unidas (ONU). Nessa última, o ONU incluiu a meta de redução da mortalidade por DCNT em um terço até 2030^15^. Monitorar as desigualdades na distribuição da mortalidade por esse grupo de causas torna-se importante no contexto dos Objetivos de Desenvolvimento Sustentável (ODS)^15^.

No Brasil, o Sistema de Informação sobre Mortalidade (SIM) possibilita acompanhar os padrões de mortalidade por essas causas e sua evolução ao longo do tempo^16^, constituindo-se como uma imprescindível fonte de dados nacional. Entretanto, problemas relativos ao preenchimento das declarações de óbito (DO) — instrumentos que alimentam o SIM — resultam em prejuízo à qualidade da informação de mortalidade. Embora se tenha observado melhora na cobertura do SIM e no preenchimento da DO nas últimas décadas, ainda se faz necessária a adoção de métodos para correção de subregistro de óbitos, de dados faltantes e do registro excessivo de causas *garbage* (CG) — que não podem ser consideradas causas básicas de óbito —, de forma a minimizar vieses nas estimativas de mortalidade^2^.

Para além do monitoramento da mortalidade prematura por DCNT na escala nacional, é preciso acompanhar a evolução desse evento em pequenas áreas — geográficas ou relativas a subgrupos populacionais. Estimativas confiáveis para pequenas áreas viabilizam um diagnóstico situacional mais detalhado e contribuem para a identificação de iniquidades em saúde, e ainda apoiam o planejamento e a priorização de ações de saúde pública^17^.

Este é o primeiro estudo que aplica método de redistribuição de causas *garbage* para gerar estimativas de mortalidade em capitais brasileiras e em diferentes estratos de privação, buscando qualificar a base de dados do SIM.

Em face do exposto, este estudo objetivou analisar a mortalidade prematura por doenças crônicas não transmissíveis nas capitais brasileiras e Distrito Federal (DF) após redistribuição das causas *garbage,* e a evolução temporal segundo estratos de privação social nos triênios 2010 a 2012 e 2017 a 2019.

## MÉTODOS

### Delineamento do estudo, população e período

Trata-se de um estudo epidemiológico descritivo. Foram analisadas estimativas de mortalidade prematura por DCNT nas capitais brasileiras e no DF referentes aos triênios 2010 a 2012 e 2017 a 2019.

### Indicadores e fonte dos dados

#### Taxa de mortalidade prematura por DCNT

Para o cálculo das taxas de mortalidade foram utilizados dados de óbitos provenientes do Sistema de Informação sobre Mortalidade. A base foi fornecida pelo Ministério da Saúde sob demanda dos pesquisadores e contém a informação dos setores censitários das residências dos falecidos. As estimativas de população, disponibilizadas no site do Datasus^18^, foram utilizadas como denominador no cálculo das taxas de mortalidade.

Foram considerados, segundo definição da OMS, os óbitos prematuros em DCNT, que compreendem os óbitos por doenças cardiovasculares, doenças respiratórias crônicas, neoplasias e diabetes *mellitus* na população com idade entre 30 e 69 anos^4,14^.

As taxas de mortalidade calculadas para cada capital e ano foram padronizadas por idade, pelo método direto^18^. Visando eliminar os efeitos das diferenças entre as estruturas etárias, adotou-se, como população padrão, a população brasileira do censo demográfico de 2010^18,19^.

No cálculo das taxas foi utilizado, no numerador, o número de óbitos estimado pelas taxas padronizadas para a idade e, no denominador, a população total dos triênios para cada capital.

#### Índice de privação social

O Índice Brasileiro de Privação (IBP), lançado em 2020 e desenvolvido por pesquisadores do Centro de Integração de Dados e Conhecimentos para Saúde (CIDACS) e da Universidade de Glasgow, na Escócia, constitui proposta para medir desigualdades sociais no Brasil na escala dos setores censitários. O cálculo do IBP considerou a combinação de escores-z de três indicadores de privação na escala de setores censitários: percentual de domicílios com renda menor que meio salário mínimo; percentual de indivíduos com sete anos de idade ou mais não alfabetizados; e percentual de indivíduos com acesso inadequado à água, esgotamento sanitário, coleta de resíduos e sem banheiro^20^.

O IBP gera um escore final em que o menor valor representa a área com menor privação, enquanto o maior valor representa a área com maior privação^20,21^. Ressalta-se que os 65.687 setores censitários das 26 capitais e do DF abrigam 45.980.851 pessoas, o correspondente a 22% de todos os setores censitários e a 24% da população brasileira. Observou-se maior concentração de privação/pobreza em capitais do Norte e Nordeste, e ausência de setores censitários de elevada privação nas regiões Sul, Sudeste e Centro-Oeste. Em razão desta distribuição desigual de quintis de setores censitários com elevada privação nas capitais, foram utilizados apenas três estratos de privação no atual estudo: O estrato de “baixa privação”, que corresponde ao quintil 1; “média privação”, que corresponde ao quintil 2; e “alta privação”, correspondente aos quintis 3, 4 e 5.

### Tratamento dos dados de óbito previamente ao cálculo das taxas de mortalidade

Considerando a heterogeneidade da qualidade do SIM no país e ao longo do tempo, foram aplicados métodos visando padronizar a qualidade das bases de dados das capitais, os quais abordaram os dados faltantes e a qualidade da definição das causas básicas de morte.

A primeira etapa consistiu no tratamento dos dados faltantes, por meio da redistribuição proporcional dos ignorados e em branco, segundo ano, idade, sexo e local de residência^22^. Posteriormente, foi feita a redistribuição das causas *garbage* (CG). Esse termo define um grupo de causas que não podem ser consideradas causas básicas de morte, ou seja, refere-se a causas mal definidas ou inespecíficas que são, portanto, de pouca utilidade para a saúde pública, posto que dificultam a identificação das reais doenças e agravos que desencadearam o óbito^22^. Por esse motivo, as CG devem ser detectadas e redistribuídas entre óbitos por causas específicas, de modo a melhorar a validade das análises sobre mortalidade^22,23^.

No processo de redistribuição das CG considerou-se, primeiramente, a definição dos grupos de causas de acordo com a lista do estudo *Global Burden of Disease* (GBD) 2017^24^, que abarca outras causas além do capítulo de causas mal definidas da Classificação Estatística Internacional de Doenças e Problemas Relacionados à Saúde (CID-10)^25^. Foi feita uma análise dos códigos dessa lista para identificar quais são as CG relacionadas especificamente às DCNT^22^. Foram identificados os óbitos cujas causas básicas, relativas aos grupos considerados neste estudo, correspondem a códigos especificados na CID-10 e definidos pelo estudo GBD 2017, classificados como causas-alvo, ou causas para as quais serão redistribuídas as CG. Posteriormente, foram feitas as redistribuições de acordo com os níveis de CG e seus respectivos alvos^22^. Além do processo de redistribuição proporcional, o estudo considerou os resultados das investigações de CG iniciadas em 2016^26^.

### Procedimentos analíticos

Foram calculados os intervalos de confiança de 95% das taxas de mortalidade prematura por DCNT. Comparações por meio da variação percentual relativa (VPR) foram feitas entre os dois triênios (2010 a 2012 e 2017 a 2019) e entre as taxas com dados antes e após a redistribuição de CG, que visa mostrar o impacto do método no risco de morte estimado pelo SIM. Todas as análises foram feitas segundo capitais e respectivas categorias do IBP.

Os setores censitários, referentes aos locais de residência dos indivíduos falecidos, foram utilizados como variável chave para atribuir a classificação de privação desses indivíduos segundo estratos do IBP. Destaca-se que os óbitos sem informação de endereço no SIM foram excluídos da análise pela impossibilidade de identificação do setor censitário e, consequentemente, de classificação em uma das categorias do IBP. A avaliação da desigualdade na distribuição da mortalidade foi feita pela análise comparativa dos valores das taxas e da variação percentual entre os estratos de IBP (baixa, média e elevada privação).

### Aspectos éticos

Esta pesquisa obedece à Resolução n^o^ 466, de 12 de dezembro de 2012, do Conselho Nacional de Saúde (CNS). Foi aprovada pelo Comitê de Ética em Pesquisa (CEP) da UFMG sob parecer de número 3.258.076.

## RESULTADOS

No geral, não foi possível geocodificar cerca de 30% dos óbitos. Essa perda foi maior no primeiro triênio em algumas capitais do Nordeste, como Fortaleza, João Pessoa e Salvador. No segundo triênio, o percentual de perdas foi menor, de 28% em média (Figura S1 material suplementar).

Considerando o conjunto das capitais no triênio de 2010 a 2012, as taxas de mortalidade por DCNT antes da redistribuição de CG foram equivalentes a 202,9 no estrato de baixa privação do IBP, 258,4 no estrato de média privação e 249,6 por 100 mil habitantes no estrato de alta privação. Após os ajustes e a redistribuição de CG, os valores das taxas aumentaram para 220,5, 285,5 e 279,4 por 100 mil habitantes nos estratos de baixa, média e alta privação, nessa ordem (Tabela 1). Houve, portanto, acréscimo de 8,7, 10,5 e 11,9%, respectivamente (Tabela S1 material suplementar).

**Tabela 1:** Taxas de mortalidade prematura por doenças crônicas não transmissíveis padronizadas por idade, por 100 mil habitantes, antes e após a redistribuição de causas *garbage*, segundo categorias do Índice Brasileiro de Privação. Capitais do Brasil, 2010 a 2012.

Regiões	Capitais	Taxa de mortalidade prematura por DCNT					
		Sem redistribuição de CG			Com redistribuição de CG		
		Baixa	Média	Alta	Baixa	Média	Alta
**Total**		**202,9**	**258,4**	**249,6**	**220,5**	**285,5**	**279,4**
Centro-Oeste	Distrito Federal	135,2	217,1	179,7	143,4	232,9	194,3
	Campo Grande	224,9	280,7	309,9	235,6	291,9	324,3
	Cuiabá	214,9	248,9	262,9	227,8	266,9	286,1
	Goiânia	166,2	222,9	259,5	182,3	243,6	284,9
Nordeste	Aracaju	145,6	203,8	240,9	159,6	222,9	271,3
	Fortaleza	97,5	120,4	126,5	105,0	133,4	142,8
	João Pessoa	128,7	186,0	184,2	138,0	200,7	206,8
	Maceió	145,9	185,4	239,9	155,8	201,3	264,4
	Natal	150,8	234,0	259,7	159,7	243,3	276,4
	Recife	178,3	244,7	322,2	185,9	257,1	341,8
	Salvador	115,5	181,2	220,9	127,1	211,2	260,9
	São Luís	156,3	158,6	219,6	165,1	170,5	232,7
	Teresina	134,7	180,0	205,5	144,9	190,9	223,9
Norte	Belém	154,6	193,5	209,2	171,9	219,4	244,9
	Boa Vista	164,7	222,9	227,3	189,0	258,5	253,9
	Macapá	212,4	150,6	149,1	238,5	175,3	183,4
	Manaus	171,7	223,4	239,6	192,7	261,2	284,7
	Palmas	173,7	199,1	226,2	182,9	208,5	237,7
	Porto Velho	183,8	205,6	216,9	203,6	231,9	249,9
	Rio Branco	155,1	162,2	220,2	168,7	175,2	252,1
Sudeste	Belo Horizonte	163,2	233,1	244,9	184,8	270,9	287,4
	Rio de Janeiro	234,1	304,3	297,6	266,6	356,3	353,0
	São Paulo	233,5	300,1	306,0	248,8	324,2	334,1
	Vitória	145,4	311,8	291,7	151,5	332,8	314,4
Sul	Curitiba	229,1	299,6	329,5	238,4	313,3	345,8
	Florianópolis	187,9	222,6	264,9	197,8	233,6	291,7
	Porto Alegre	239,9	331,5	281,3	252,5	353,6	303,9

DCNT: doenças crônicas não transmissíveis; CG: causas *garbage*.

Esse padrão de taxas mais altas após a redistribuição de CG foi observado em todas as capitais no primeiro triênio e, em geral, as taxas foram mais altas também no estrato de mais alta privação. Após a redistribuição de CG, o acréscimo nas taxas das áreas intraurbanas com baixa privação variou de 4,1% (Curitiba) a 14,8% (Boa Vista); nas áreas com média privação, variou de 4,0% (Natal) a 17,1% (Rio de Janeiro) e, no estrato de alta privação, variou de 4,6% (Campo Grande) a 23,0% (Macapá) (Tabela S1 material suplementar).

Ainda no primeiro triênio, as taxas após a redistribuição de CG no estrato de baixa privação variaram de 105,0 em Fortaleza a 266,6 por 100 mil habitantes no Rio de Janeiro. No estrato de média privação, Fortaleza apresentou a taxa mais baixa, de 133,4 por 100 mil habitantes e Rio de Janeiro a mais alta, de 356,3 por 100 mil. No estrato de alta privação, a taxa mais baixa ocorreu em Fortaleza e a mais alta no Rio de Janeiro, respectivamente, 142,8 e 353 por 100 mil habitantes (Tabela 1 e Tabela S1 material suplementar).

No triênio 2017 a 2019, as taxas de mortalidade antes da redistribuição de CG corresponderam a 163,9 no estrato de baixa privação, 224,5 média privação e 231,9 por 100 mil habitantes no de alta privação (Tabela 2). As taxas aumentaram após a redistribuição de CG, passando para 180,3, 248,7 e 258,4 por 100 mil habitantes nesses estratos, acréscimo de 10, 10,8 e 11,4%, respectivamente (Tabela 2 e Tabela S2 material suplementar).

**Tabela 2:** Taxas de mortalidade prematura por doenças crônicas não transmissíveis padronizadas por idade, por 100 mil habitantes, antes e após a redistribuição de causas *garbage*, segundo categorias do Índice Brasileiro de Privação. Capitais do Brasil, 2017 a 2019.

Regiões	Capitais	Total DCNT					
		Sem redistribuição de CG			Com redistribuição de CG		
		Baixa	Média	Alta	Baixa	Média	Alta
**Total**		**163,9**	**224,5**	**231,9**	**180,3**	**248,7**	**258,4**
Centro-Oeste	Distrito Federal	97,4	175,6	160,5	101,9	184,7	170,3
	Campo Grande	180,2	244,7	305,4	190,1	257,7	321,3
	Cuiabá	178,4	228,2	268,3	186,7	235,8	280,2
	Goiânia	154,2	199,7	261,6	160,1	208,9	272,9
Nordeste	Aracaju	127,4	160,8	207,4	138,5	177,9	232,8
	Fortaleza	95,1	133,3	139,4	103,7	145,9	152,4
	João Pessoa	122,1	144,8	152,4	130,1	158,2	170,6
	Maceió	131,2	156,9	216,9	139,8	168,9	236,7
	Natal	142,3	220,4	255,1	148,9	234,6	274,7
	Recife	151,1	225,1	307,6	156,6	234,4	322,7
	Salvador	105,0	164,5	202,8	118,2	188,9	240,5
	São Luís	98,9	123,2	164,1	109,7	132,4	178,1
	Teresina	130,9	144,6	185,2	138,9	155,5	197,7
Norte	Belém	128,3	177,9	192,6	140,1	191,6	210,8
	Boa Vista	193,9	258,9	297,7	205,2	272,5	311,2
	Macapá	184,1	205,9	198,8	218,8	244,1	234,9
	Manaus	135,1	171,0	197,4	157,2	203,1	238,0
	Palmas	128,3	127,8	153,6	132,2	132,9	158,3
	Porto Velho	145,3	179,8	210,6	166,5	208,9	241,9
	Rio Branco	180,2	179,8	226,8	213,8	199,1	261,8
Sudeste	Belo Horizonte	128,5	207,7	241,9	143,5	233,7	272,2
	Rio de Janeiro	213,2	295,5	293,3	243,8	345,9	348,2
	São Paulo	183,4	254,2	281,2	200,3	280,2	310,6
	Vitória	124,9	285,6	273,4	128,9	295,0	279,4
Sul	Curitiba	141,9	176,9	189,6	149,9	186,4	203,9
	Florianópolis	166,4	252,9	273,4	175,9	267,3	290,5
	Porto Alegre	186,5	294,7	249,4	205,0	318,1	271,6

DCNT: doenças crônicas não transmissíveis; CG: causas *garbage*.

Em geral, as taxas aumentaram nas capitais após a redistribuição de CG e foram maiores nos estratos de alta privação. A variação percentual foi mais baixa em Vitória, em comparação com as demais capitais, nos três estratos de privação: 3,2, 3,3 e 2,2% nos setores censitários de baixa, média e alta privação, respectivamente. A variação foi mais elevada em Macapá nos mesmos estratos: 18,8, 18,6 e 18,2% (Tabela S2 material suplementar).

No estrato de baixa privação, as taxas de mortalidade após a redistribuição de CG variaram de 101,9 no Distrito Federal a 243,8 por 100 mil habitantes no Rio de Janeiro. No estrato de média privação, as taxas variaram de 132,4 em São Luís a 345,9 por 100 mil habitantes no Rio de Janeiro; quanto ao estrato de alta privação, a taxa mais baixa foi em Fortaleza e a mais elevada no Rio de Janeiro: 152,4 e 348,2 por 100 mil habitantes (Tabela 2 e Tabela S2 material suplementar).

Analisando-se a evolução das taxas ajustadas ao longo dos dois triênios, observou-se redução no conjunto das capitais, em todos os estratos de privação. O maior decréscimo ocorreu no estrato de baixa privação (-18,2%), e o menor no estrato de alta privação (-7,5%) (Tabela 3). As capitais com maior redução no estrato de baixa privação foram: Curitiba (-37,1%), São Luís (-33,6%), Distrito Federal (-28,9%), Palmas (-27,7%) e Belo Horizonte (-22,3%). No estrato de média privação, os maiores decréscimos ocorreram em Curitiba (-40,5%) e Palmas (-36,3%), e no estrato de alta privação os decréscimos mais expressivos ocorreram em Curitiba (-41,0%), Palmas (-33,4%) e São Luís (-23,5%). Duas capitais apresentaram acréscimo das taxas nos três estratos de privação: Rio Branco (26,7, 13,6, 3,8%) e Boa Vista (8,6, 5,4 e 22,6%). Macapá (39,2 e 28,1%) e Fortaleza (9,4 e 6,7%) tiveram aumento nos estratos de média e alta privação (Tabela 3).

**Tabela 3:** Variação percentual da mortalidade prematura por doenças crônicas não transmissíveis entre os triênios 2010-2012 e 2017-2019, segundo Índice Brasileiro de Privação, após a redistribuição de causas *garbage* nas capitais.

Regiões	Capitais	Variação percentual (%) IBP		
		Ba	Me	Al
**Total**		**−18,2**	**−12,9**	**−7,5**
Centro-Oeste	Distrito Federal	−28,9	−20,7	−12,4
	Campo Grande	−19,3	−11,7	−0,9
	Cuiabá	−18,0	−11,7	−2,1
	Goiânia	−12,2	−14,2	−4,2
Nordeste	Aracaju	−13,2	−20,2	−14,2
	Fortaleza	−1,2	9,4	6,7
	João Pessoa	−5,7	−21,2	−17,5
	Maceió	−10,3	−16,1	−10,5
	Natal	−6,8	−3,6	−0,6
	Recife	−15,8	−8,8	−5,6
	Salvador	−7,0	−10,6	−7,8
	São Luís	−33,6	−22,3	−23,5
	Teresina	−4,1	−18,5	−11,7
Norte	Belém	−18,5	−12,7	−13,9
	Boa Vista	8,6	5,4	22,6
	Macapá	−8,3	39,2	28,1
	Manaus	−18,4	−22,2	−16,4
	Palmas	−27,7	−36,3	−33,4
	Porto Velho	−18,2	−9,9	−3,2
	Rio Branco	26,7	13,6	3,8
Sudeste	Belo Horizonte	−22,3	−13,7	−5,3
	Rio de Janeiro	−8,6	−2,9	−1,4
	São Paulo	−19,5	−13,6	−7,0
	Vitória	−14,9	−11,4	−11,1
Sul	Curitiba	−37,1	−40,5	−41,0
	Florianópolis	−11,1	14,4	−0,4
	Porto Alegre	−18,8	−10,0	−10,6

IBP: Índice Brasileiro de Privação; Ba: baixa privação; Me: média privação; Al: alta privação; DCNT: doenças crônicas não transmissíveis.

## DISCUSSÃO

O estudo mostrou o impacto da correção de dados de óbito na estimativa das taxas de mortalidade prematura por DCNT. Observou-se que as taxas aumentaram entre 8 e 12% após a redistribuição de CG, e os maiores acréscimos ocorreram nas áreas mais vulneráveis. Ao considerar os triênios iniciais e finais, ocorreram importantes decréscimos da mortalidade na maioria das capitais brasileiras, sendo os mais expressivos nos estratos de baixa vulnerabilidade. Reduções acima de 20% entre os triênios, em todos os extratos de vulnerabilidade, foram observadas em Curitiba, São Luís e Palmas. Por outro lado, incrementos nas taxas das capitais Rio Branco, Boa Vista, Macapá e Fortaleza foram observados no último triênio.

O SIM foi criado para a obtenção regular de dados sobre mortalidade no país e apresenta cobertura universal. Avanços relacionados à ampliação da cobertura dos óbitos, completude de dados e à melhor definição das causas básicas de morte, por meio da diminuição da proporção de CG^2^, comprovam os bons resultados de ações do Ministério da Saúde para qualificação dos dados de mortalidade no país. No entanto, o tratamento desses dados se mostra importante, em especial, nas análises comparativas espaço-temporais, uma vez que a qualidade dessas informações é heterogênea no país, no qual as regiões Norte e, sobretudo, Nordeste ainda representam os piores cenários^26^.

Nesse sentido, o uso do dado bruto requer atenção, e recomenda-se que sejam empregados tratamentos na base do SIM antes da realização de análises de situação de saúde, principalmente em âmbito subnacional^16,23^. Recomenda-se a correção de óbitos subregistrados, bem como a consideração das causas mal definidas e dos CG por meio de métodos de redistribuição^16,23^. O presente estudo reforça a importância de se adotar esses métodos ao avaliar o impacto nas taxas antes e após o tratamento dos dados de óbito em pequenas áreas.

O estudo inova ao apresentar dados corrigidos com metodologia que considera a redistribuição de CG. A metodologia proposta por Teixeira et al.^22^ introduz algoritmos de redistribuição de CG específicos para o Brasil, uma vez que considera os resultados de investigações de óbitos iniciado no projeto com 60 cidades^2^. Ao propor essa metodologia empírica baseada nas investigações realizadas nas secretarias estaduais de saúde, a metodologia considera o contexto brasileiro que é diferente do observado em outros países^23,27^.

Analisando-se as estimativas após tratamento dos dados, um achado importante consistiu na redução das taxas de mortalidade por DCNT na maioria das capitais brasileiras, em especial nos estratos de menor vulnerabilidade. Malta et al.^5^, ao analisarem dados do GBD entre 1990 e 2017, identificaram declínio de 35,9% na mortalidade por DCNT prematura, sendo que as doenças cardiovasculares tiveram a redução mais expressiva, de 47,9%. Esses avanços podem ser explicados pela melhoria das condições de vida e saúde; redução da pobreza; maior acesso a bens e serviços; e expansão do Sistema Único de Saúde (SUS) com aumento da cobertura do seu rol de serviços, bem como da atenção primária, o que possibilitou atender as demandas da população brasileira impulsionadas pelas transições epidemiológica, nutricional e demográfica^28^. Somam-se a isso os avanços nas políticas de saúde, incluindo a expansão do Programa Saúde da Família; implementação do Plano de Ações Estratégicas para o Enfrentamento das DCNT^7,13^; a Política Nacional de Promoção da Saúde^29^; e implementação de medidas regulatórias, como a lei de ambientes livres de tabaco^30^, entre outras.

Por outro lado, algumas capitais do Norte e do Nordeste apresentaram incremento das taxas entre os triênios, o que pode ser explicado pela melhora na qualidade das informações de mortalidade nas últimas décadas e avanços relacionados à ampliação da cobertura dos óbitos, à notificação mais precisa sobre as causas e à diminuição da proporção de CG^2,27^.

As diferenças nas estimativas das taxas por DCNT entre as categorias do IBP, com taxas mais elevadas na maioria das capitais e em ambos os triênios nos estratos de maior privação, é um resultado importante no que se refere à avaliação de desigualdades em saúde na escala intraurbana. Essas diferenças têm sido bem documentadas na literatura, que sugere que a população de baixa renda e menor escolaridade detém taxas mais elevadas de DCNT^8^ e de fatores de risco para essas doenças^31,32^ em razão das desigualdades sociais e baixo acesso a bens e serviços de saúde^8^. Nesse sentido, análise da Pesquisa Nacional de Saúde (PNS) 2013 sobre as prevalências dos comportamentos de saúde da população brasileira revelou profundas desigualdades sociais: os menos escolarizados, pardos ou pretos e aqueles sem plano privado de saúde apresentaram maior prevalência de tabagismo, inatividade física no lazer, sedentarismo, consumo de leite integral e baixo consumo de verduras, vegetais e frutas^31^. Além disso, os indivíduos pretos ou pardos apresentaram maior prevalência do consumo excessivo de álcool em comparação aos brancos^32^.

O aumento da carga das DCNT acarreta desafios para a saúde pública brasileira. Além de afetarem de forma desigual as populações, ao impactarem mais negativamente aquelas em situação mais vulnerável do ponto de vista social e econômico, as DCNT representam um obstáculo para o desenvolvimento socioeconômico^12^. A despeito dos avanços no enfrentamento das DCNT, a meta de reduzir em 30% a mortalidade prematura por DCNT até 2030 não deverá ser alcançada^5^. Para tanto, entende-se como necessário avançar com ação coordenada entre os governos federal, estaduais e municipais, orientada a ampliar e melhorar a capacidade de oferta e de acesso aos serviços de saúde; a adotar medidas regulatórias de controle de alimentos não saudáveis, tabaco, álcool, entre outros fatores de risco; e medidas de prevenção e de tratamento^3,5,14^.

O estudo apresenta algumas limitações. Entre elas, a utilização do IBP, que foi construído com dados do censo de 2010 e pode não captar as mudanças na distribuição da privação na última década. Isso só poderá ser aferido após o censo demográfico de 2022. Além disso, considerando o conjunto das capitais, 30% dos óbitos, em média, não puderam ser geocodificados e, portanto, não foram classificados nas categorias do IBP. Destaca-se que as taxas de mortalidade podem ser ainda mais elevadas nas áreas de maior privação, em função de maior proporção de óbitos não georreferenciados nessas áreas. As análises se limitaram às capitais em razão da melhor qualidade da informação de óbito nessas localidades. Isso reflete na produção de estimativas com maior acurácia. Como fortaleza, o estudo inova nos avanços metodológicos, como o tratamento dos dados faltantes e a redistribuição das causas *garbage*, contribuindo com o *corpus* de pesquisas sobre a melhoria das estimativas de mortalidade no país. Ademais, pela primeira vez utilizou-se o IBP para calcular as desigualdades nas estimativas de mortalidade por DCNT nas capitais.

Em suma, o presente estudo evidenciou que a redistribuição de CG representou aumento dos valores das taxas de mortalidade por DCNT dos municípios investigados, e o incremento foi maior nos estratos de maior privação social. Observou-se taxas de mortalidade mais elevadas nos estratos de maior privação social. Na análise da evolução temporal, ocorreu decréscimo da mortalidade prematura por DCNT, sobretudo em áreas de menor privação social.

O uso de metodologia para tratamento dos dados de mortalidade está em constante desenvolvimento, entretanto é importante incentivar a melhoria da vigilância e do preenchimento das declarações de óbito, e reforçar o treinamento e a educação continuada de profissionais que lidam com a notificação de óbitos.
